# Synthesis and Preliminary Investigations of the siRNA Delivery Potential of Novel, Single-Chain Rigid Cationic Carotenoid Lipids

**DOI:** 10.3390/molecules17033484

**Published:** 2012-03-16

**Authors:** Michael D. Pungente, Emile Jubeli, Christer L. Øpstad, Mais Al-Kawaz, Nour Barakat, Tarek Ibrahim, Nada Abdul Khalique, Liji Raju, Rachel Jones, Philip L. Leopold, Hans-Richard Sliwka, Vassilia Partali

**Affiliations:** 1 Pre-Medical Unit, Weill Cornell Medical College in Qatar, Doha, P.O. Box 24144, Qatar; 2 Research Division, Weill Cornell Medical College in Qatar, Doha, P.O. Box 24144, Qatar; 3 Department of Chemistry, Norwegian University of Science and Technology (NTNU), 7491 Trondheim, Norway; 4 Department of Chemistry, Chemical Biology & Biomedical Engineering, Stevens Institute of Technology, Hoboken, NJ 07030, USA

**Keywords:** carotenoid lipids, single-chain, rigid, cationic, non-viral siRNA delivery

## Abstract

The success of nucleic acid delivery requires the development of safe and efficient delivery vectors that overcome cellular barriers for effective transport. Herein we describe the synthesis of a series of novel, single-chain rigid cationic carotenoid lipids and a study of their preliminary *in vitro* siRNA delivery effectiveness and cellular toxicity. The efficiency of siRNA delivery by the single-chain lipid series was compared with that of known cationic lipid vectors, 3β-[*N*-(*N'*,*N'*-dimethylaminoethane)carbamoyl]-cholesterol (DC-Chol) and 1,2-dimyristoyl-*sn*-glyceryl-3-phosphoethanolamine (EPC) as positive controls. All cationic lipids (controls and single-chain lipids) were co-formulated into liposomes with the neutral co-lipid, 1,2-dioleolyl-*sn*-glycerol-3-phosphoethanolamine (DOPE). Cationic lipid-siRNA complexes of varying (+/−) molar charge ratios were formulated for delivery into HR5-CL11 cells. Of the five single-chain carotenoid lipids investigated, lipids **1**, **2**, **3** and **5** displayed significant knockdown efficiency with HR5-CL11 cells. In addition, lipid **1** exhibited the lowest levels of cytotoxicity with cell viability greater than 80% at all (+/−) molar charge ratios studied. This novel, single-chain rigid carotenoid-based cationic lipid represents a new class of transfection vector with excellent cell tolerance, accompanied with encouraging siRNA delivery efficiency.

## 1. Introduction

Cationic lipids are promising non-viral vector systems for use in small interfering RNA (siRNA) and DNA delivery [1,2,3,4] to effect the introduction of exogenous sequences of DNA into cells to correct defective genes [5,6,7] or to selectively silence gene expression, referred to as RNA interference, or RNAi, through siRNA delivery [8,9,10,11]. Over the past several years, novel cationic lipid vectors have been synthesized and combined with nucleic acids for these purposes [12,13].

In order for the lipid-nucleic acid complex (or lipoplex) to cross the cell membrane, the complex should be charge-neutral or have an excess positive charge overall. The use of cationic lipids facilitates lipoplex formation by developing a charge-neutral complex with the negatively charged nucleic acid (DNA or siRNA). Although unsolved, the mechanism by which the lipid/nucleic acid complex is internalized into the cell is thought to occur by endocytosis. The lipoplex size is important for active endocytosis [14]. Larger particles may have better initial contact with the cells [15], and increased phagocytic activity accompanied by endosomal escape [16]. Lipoplexes greater than 250 nm in diameter result in larger endosomes that are more easily ruptured [17]. It should be mentioned that large particles on the order of the size of a cell are inefficient for *in vivo* administration as they become trapped in the capillary regions.

**Figure 1:**
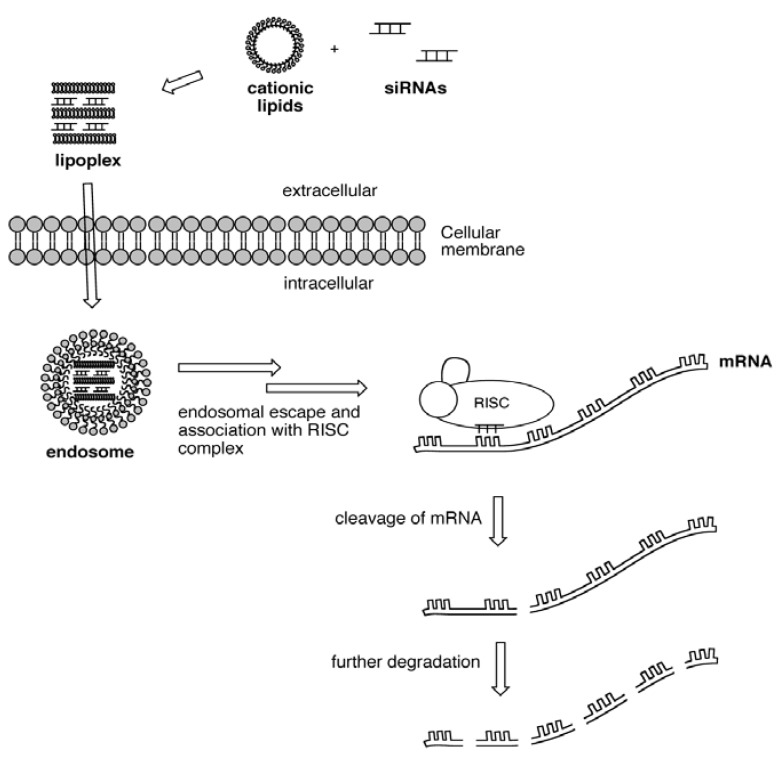
Delivery of lipid-siRNA complex leading to cleavage of mRNA.

In the case of siRNA delivery, the lipoplex must escape the endosome and traffic the cytoplasm where the siRNA is taken up by the RNA-induced silencing complex (RISC), leading ultimately to the catalytic destruction of a complimentary endogenous messenger RNA (mRNA), as illustrated in Figure 1. This results in preventing the native mRNA from producing a protein product; this process is referred to as “knockdown”. However, knockdown is not without restrictions when it comes to practical applications. The clinical application of RNAi is restrained by lack of tissue specificity, degradation of the complex by cellular components, and toxicity associated with the cationic lipid carrier.

**Figure 2:**
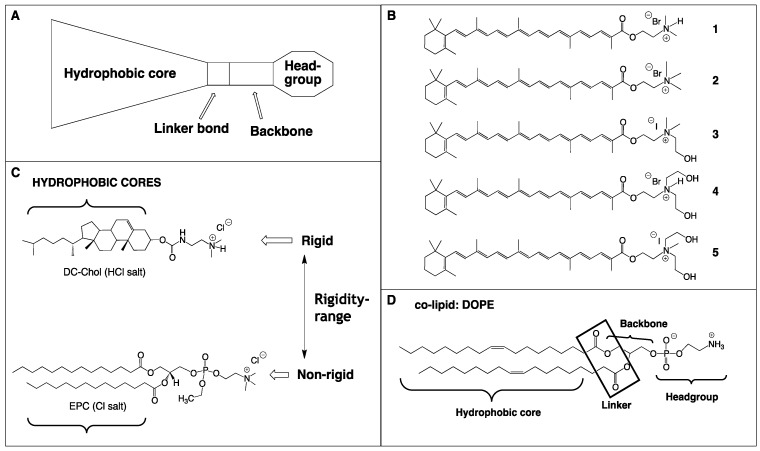
The basic structural of components used in this study. **A**: General lipid structure including a hydrophobic domain, linker bond, backbone and a polar headgroup; **B**: Structures of single-chain rigid cationic carotenoid lipids, **1**–**5**; **C**: The rigidity range associated with the hydrophobic domain of common cationic lipids, DC-Chol and EPC used as positive controls; **D**: Structure of co-lipid DOPE.

Current commercially available cationic glycerolipids used for siRNA delivery are not effective enough as siRNA delivery vectors. These lipids are characterized by a common structural motif (Figure 2A) that includes a hydrophilic headgroup, linker bond, backbone (typically glycerol) and two hydrophobic tails, mainly as saturated fatty acid chains. We believe that structural modifications to the headgroup and the hydrophobic core of lipid vectors are key conditions to enhancing siRNA delivery.

Nucleic acid binding by the cationic lipid vector calls for a headgroup that can sustain a positive charge at physiological pH. Typical headgroup moieties to achieve this include primary, secondary, or tertiary amines, and in addition, quaternary ammonium salts, guanidine, and imidazole groups have been successfully employed [18]. A large number of cationic lipids are functionalized with polyamine headgroups, where spermine and spermidine groups are very common. Some reports suggest that since formulations using polyvalent lipids require a lower stoichiometric amount of cationic lipid to reach charge neutrality with the negative charges associated with the phosphate groups of the nucleic acid, such polyvalent lipids are typically less toxic [19]. A risk associated with the polyvalent cationic headgroup is that the electrostatic interaction between the lipid and nucleic acid cargo is too intense, resulting in failure to release the cargo to allow the intended function of the genetic material. Non-viral lipid vectors functionalized with the quaternary amine headgroup are reported to be more toxic than those containing the tertiary amine headgroup [20].

Rigid lipids have been shown to self-assemble into tightly packed vesicles [21]. Such findings have lead us to the following two hypotheses: First, that the packing or self-assembling characteristics of our novel, single-chain rigid cationic lipids, **1**–**5** (Figure 2B) should be more related to the self-assembling characteristics of other rigid lipids, such as DC-Chol, and less like non-rigid lipids, for example EPC (Figure 2C). Secondly, that the lipid-siRNA lipoplexes generated from our rigid carotenoid lipids and those generated with the rigid control lipid, DC-Chol, would ultimately give rise to a similar therapeutic siRNA performance, but dissimilar to the non-rigid vector, EPC; hence the choice of our two positive control lipids.

We have reduced the complexity of glycerolipids by synthesizing lipid-like compounds (lipidoids) in which the non-rigid, saturated fatty chain is replaced by a rigid, polyunsaturated fatty acid directly esterified with aminoethanol derivatives.

Herein we report, to our knowledge, the first use of cationic carotenoid lipids as siRNA delivery vectors. Our results revealed that the single-chain, rigid carotenoid lipids **1**, **2**, **3** and **5** are effective siRNA vectors for *in vitro* knockdown of luciferase activity in the HR5-CL11 cell line. Furthermore, lipid **1** displayed comparable cell tolerance to EPC, and far less toxicity than DC-Chol.

## 2. Results

Synthetic vectors for transgene therapy have the advantage over viral vectors of being more biocompatible, having a lower immunogenic effect with simplicity of production and application [22]. Current commercially available cationic lipids used for siRNA delivery have a common structural motif that includes a hydrophilic headgroup linked typically to two non-rigid (flexible) hydrophobic tails. Herein, five novel lipids, **1**–**5** having a common rigid C30-carotenoid hydrophobic domain, while differing in the nature of the amphiphilic headgroups, were synthesized and evaluated for liposome and lipoplex formation. In addition, these cationic carotenoid lipids were evaluated for their effectiveness to deliver a specific GL2 siRNA to effect knockdown of luciferase activity in the HR5-CL11 cell line in cell culture, compared to control lipids DC-Chol and EPC.

### 2.1. Synthesis of Single-Chain Cationic Carotenoid Lipids, *1–5*

**Scheme 1:**
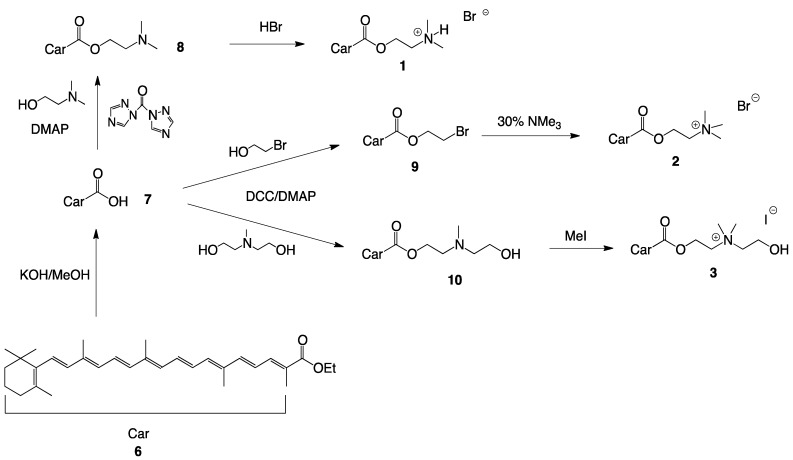
Synthesis of single-chain cationic carotenoid lipids **1**–**3**.

**Scheme 2:**
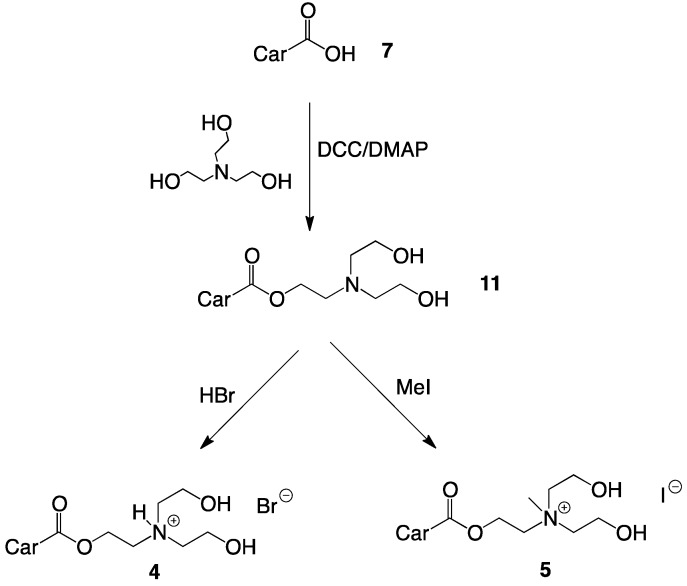
Synthesis of single-chain cationic carotenoid lipids **4** and **5**.

Cationic choline-ester analogues were prepared in high yields from *β*-apo-8'-carotenoic acid (**7**), obtained by alkaline hydrolysis of ethyl-*β*-apo-8'-carotenoate (**6**) (Scheme 1 and Scheme 2) [23].

Esterifications were performed with dicyclohexylcarbodiimide (DCC) and 4-dimethylamino pyridine (DMAP) and bromoethanol with subsequent amination or directly with the appropriate aminoethanol derivatives. The alcohols were used in excess to ensure rapid transformation. After reaction, alcohol and the urea by-product from DCC were removed by extraction and precipitation from cold acetone, respectively. The aminoethanol esters were purified by flash-column chromatography (CC) and recrystallized from acetone.

HBr salts were prepared by addition of aqueous HBr to the neutral amines with subsequent freeze-drying and recrystallization in acetone. The aminoethanol esters were quaternized with excess methyl iodide in THF. The crude salts were isolated by filtration and recrystallized in acetone.

### 2.2. Lipid/siRNA Lipoplex Formulation and Particle Sizing

Our work was inspired by the hypothesis that single-chain, rigid cationic lipids would tightly self-assemble around negatively charged nucleic acids, resulting in stable lipoplexes that perform knockdown as effectively, or better than the known rigid cationic vector, DC-Chol. Liposome particle sizing was the initial study to assess the self-assembling characteristics of our novel, single-chain rigid cationic lipids, **1**–**5**, together with the known rigid vector, DC-Chol, and non-rigid lipid EPC.

**Figure 3:**
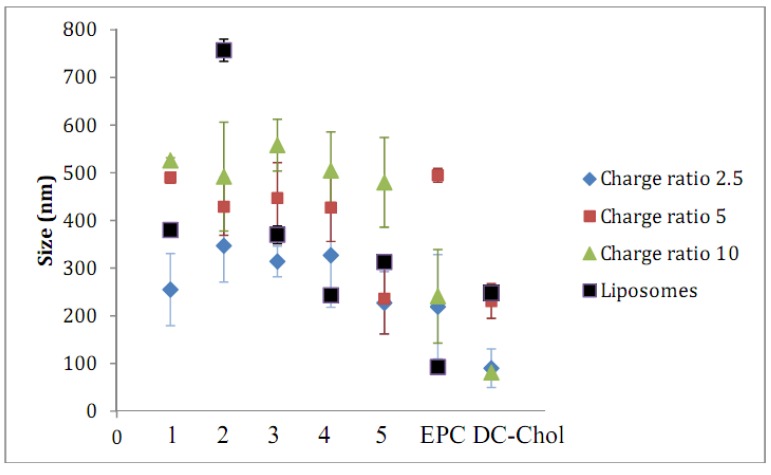
Liposome and lipid-siRNA lipoplex particle sizes were determined by dynamic light scattering at 25 °C at a detection angle of 90°. Separate hydrated liposome solutions (from sterile water) composed of cationic lipid/DOPE (3:2 mole/mole ratio) were generated in duplicate for each carotenoid lipid, **1**–**5**, as well as for control lipids EPC and DC-Chol, and each sample was analyzed in triplicate. The corresponding liposome-siRNA lipoplexes were prepared in OPTI-MEM^®^ buffer at (+/−) molar charge ratios 2.5, 5 and 10. All data are the mean ± standard error (S.E.) of three measurements for two different batches.

In a first step, cationic liposomes were prepared through the sonication of a hydrated thin film of lipids formed upon elimination of ethanol by rotary evaporation. The liposome particle size data obtained by dynamic light scattering (Figure 3) reveals a range in average liposome diameter between 100–400 nm for the majority of the lipids analyzed, with the exception of lipid **2**, which resulted in average liposome diameters of 757 nm. Interestingly, liposomes composed of EPC were generally smaller than all of the rigid cationic lipids in this study, including DC-Chol.

The lipid headgroup choices were based on groups common to those that are presented in the literature. The positive charge associated with headgroups composed of quaternary ammonium salts is isolated mainly on nitrogen, whereas this charge can be delocalized to the N-H bond in lower order salts. This delocalization of charge permits the surrounding water molecules to reduce the positive charge through hydrogen bonding interactions. This interaction of HBr salts (**1**- and **4**-based liposomes) with water participates in the stabilization of liposomes in the aqueous media. Liposomes prepared from lipids **3**–**5** containing hydroxyl moieties at the headgroups may exhibit a similar stabilizing effect through the interaction with surrounding water molecules. In contrast, lipid **2** containing a quaternary ammonium cannot participate in such stabilization, and thus gave rise to the formation of aggregates upon hydration.

Combining the negatively charged siRNA with the cationic liposomes resulted in lipoplex formation initially mediated by electrostatic interactions and subsequently by hydrophobic effects [12,24,25]. The carotenoid lipid/siRNA lipoplexes assemble into nanosized particles ranging from 100–550 nm diameters, where the smallest particles correspond to lipoplexes with a (+/−) molar charge ratio of 2.5, and largest particles at a (+/−) molar charge ratio of 10:1, as expected [26]. Aggregates ranging in size from 1 to 5 µm were detected in all lipoplex samples, particularly with lipoplexes prepared from the carotenoid lipid **2**. Only the major populations formed by the submicron size particles were taken into consideration in the calculation of lipoplex size.

### 2.3. Lipid/siRNA Transfection

The efficiency of the lipoplex-mediated siRNA delivery (the ability of lipid-transported GL2 anti-luciferase to knockdown the luciferase expression compared to that of naked GL2 alone) was investigated by a luciferase knockdown assay in HR5-CL11 cells, stably transfected with the luciferase reporter via a tetracycline controlled transcriptional trans-activator. Cells were transfected with lipoplexes for 4 h before the media was replaced with complete growth media containing antibiotics followed by incubation at 37 °C, 5% CO_2_ for 48 h before performing the assay.

**Figure 4:**
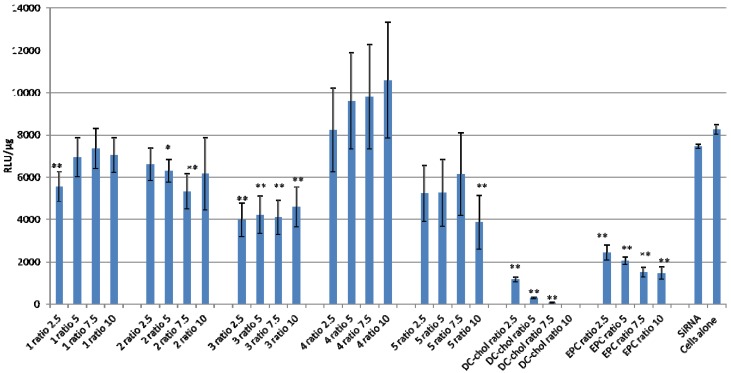
Luciferase knockdown for carotenoid lipoplex formulations, **1**–**5**, and controls DC-Chol and EPC, 48 h after transfection with various N/P (+/−) molar charge ratios. Data are expressed as relative light units (RLU)/µg of protein. Data are the average of three experiments, each performed in triplicate (n = 9). Data are expressed as mean ± S.E.* *p* < 0.05, ** *p* < 0.01 for GL2 siRNA *versus* control untreated cells (Student’s t-test).

In general, the data revealed variable knockdown performance for the different vectors assayed (Figure 4). Lipoplexes composed from lipid **4** did not result in knockdown activity, whereas those composed of carotenoid lipids **1**, **2**, **3** and **5** revealed significant anti-luciferase capacity for the GL2 anti-luciferase treated group at one or more (+/−) charge ratios studied. Lipoplexes composed from lipid **2** revealed a moderate anti-luciferase capacity at (+/−) charge ratio 5 for the GL2 anti-luciferase treated group (*p* < 0.05). Formulations that displayed significant efficiency towards luciferase knockdown in GL2 treated HR5 CL11 cells over non-treated cells (*p* < 0.01) are those containing lipid **1** at the lowest (+/−) charge ratio studied, lipid **2** with (+/−) charge ratios of 7.5, and lipid **5** at the highest (+/−) charge ratio studied. All lipoplexes containing lipid **3** formulated with (+/−) charge ratios of 2.5–10 displayed significant efficiency towards luciferase knockdown in GL2 treated HR5 CL11 cells. Cells treated with control siRNA-containing formulations revealed equal or greater luciferase signal than those treated with GL2 (data not shown). 

Although these novel carotenoid lipids revealed promising efficiencies within particular (+/−) charge ratios, none proved as efficient in knocking down luciferase expression as the control lipids, DC-Chol and EPC, two known non-viral gene delivery agents.

### 2.4. Cellular Toxicity

**Figure 5:**
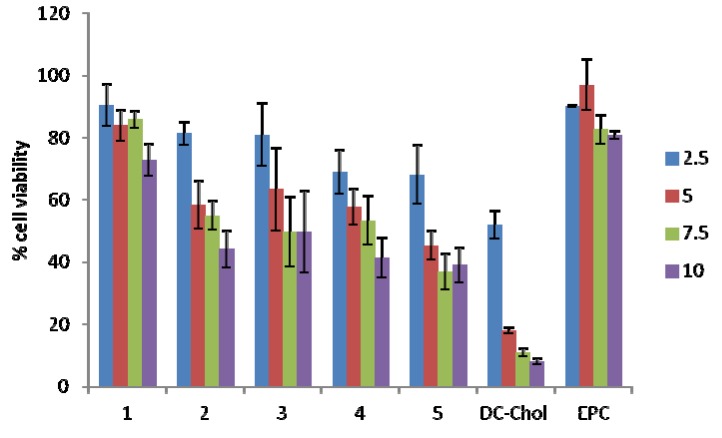
Cytotoxicity of carotenoid lipoplex formulations, **1**–**5**, and control lipids DC-Chol and EPC, 48 h after transfection with various N/P (+/−) molar charge ratios. The percentage of viable cells was calculated as the absorbance ratio of treated to untreated cells. Data for (+/−) molar charge ratios 2.5–10 are the average of three experiments (n = 9); Data are expressed as mean ± S.E.

It is well documented that the charge ratio of the positive lipid to negative nucleic acid cargo affects toxicity, with higher (+/−) charge ratios generally being more toxic [27]. The toxicity of lipoplexes may cause inflammatory reactions by interaction with the reticuloendothelial system (RES) [28,29]. The cytotoxicity associated with the carotenoid-based lipoplexes and those of DC-Chol and EPC with (+/−) molar charge ratios ranging from 2.5 to 10, was determined on the HR5-CL11 cell line after a 48 h incubation at 37 °C with 5% CO_2_ using the MTS assay. Results reported in Figure 5 reveal concentration-dependent cytotoxicity associated with all lipoplex formulations, with the exception of the carotenoid lipid **4**, and EPC.

Lipoplexes containing lipid **2**, as well as those formulated from **3**–**5** (functionalized with hydroxyl moieties at the lipid headgroup) were found to be cytotoxic beyond (+/−) molar charge ratio 5, with less than 50% cell viability. Lipoplexes containing lipid **1** and EPC were very well tolerated by the HR5 CL11 cells at all charge ratios studied. Those containing DC-Chol exhibited high cytotoxicity beyond charge ratio 2.5.

## 3. Discussion

Herein, five novel lipids having a common rigid C30-carotenoid hydrophobic domain, while differing in the nature of the amphiphilic headgroups, were synthesized and evaluated for their ability to deliver siRNA.

Lipids **1** and **2**, with tertiary and quaternary amine headgroups, respectively, revealed knockdown efficiencies at various charge ratios. Lipid **1** displayed significant knockdown only at (+/−) charge ratio 2.5 whereas lipid **2** produced significant knockdown at (+/−) charge ratios 5 and 7.5. Lipid **1** was significantly less toxic than **2** for all (+/−) charge ratios studied.

Cationic lipids containing amine headgroups functionalized with an hydroxyethyl moiety have been shown to enhance transfection efficiency [30,31], hence the justification for lipids **3**–**5**. This might explain the significant knockdown efficiency for lipid **3** at all (+/−) charge ratios, not seen with lipids **1** and **2**. Adding a second hydroxyethyl group (as is the case for lipids **4** and **5**) did not further enhance the knockdown efficiency, but rather increased the cytotoxicity (Figure 5).

One limitation of this study to note is that two of the five cationic carotenoid lipids, namely **3** and **5**, support an iodide counter ion while the remaining three carotenoid lipids have a bromide counter ion. This could potentially complicate the interpretation of results when comparing the relative knockdown efficiencies.

The luciferase expression (relative light units, RLU) was normalized by the total protein content (absorbance at 562 nm, A562) to decouple cytotoxicity from luciferase knockdown.

In Figure 4, DC-Chol appeared to exhibit a superior knockdown efficiency over all charge ratios studied, however, it was revealed through the MTS assay (Figure 5) that DC-Chol exhibited high cytotoxicity beyond charge ratio 2.5, and therefore the knockdown results may be taken with precaution as the conclusion may be more a function of cell death rather than RNA interference.

Lipids **1**, **2**, **3** and **5** (particularly **1**) combine good cell tolerance with knockdown activity and therefore represent suitable candidates for further investigation.

## 4. Experimental

### 4.1. Materials

Lipids DC-Chol and EPC were obtained from Avanti Polar Lipids (Alabaster, AL, USA). All solvents and chemicals reagents were obtained from Sigma Aldrich (St. Louis, MO, USA) unless otherwise stated. Cell culture media and antibiotics were purchased from Invitrogen Ltd. (Paisley, UK). Dichloromethane was obtained from Alfa Aesar (West Hill, MA, USA).

### 4.2. SiRNA Sequences

The HR5-CL11 cell line, a Hela derivative, was purchased from Health Protection Agency Culture Collections (Salisbury, UK). Duplex siRNA targeted to the GL2 luciferase gene (Target Sequence: 5'- CGT ACG CGG AAT ACT TCG A -3') and siGENOME non-targeting siRNAs (negative control) were both obtained from ThermoFisher Scientific (Lafayette, CO, USA).

### 4.3. General Methods

NMR spectra (^1^H, and ^13^C) were acquired on a Bruker Avance DPX 400 MHz with CDCl_3_ unless otherwise stated. UV-Vis spectra were recorded in CH_2_Cl_2_ using a Single Beam Thermo Spectronic, Helios γ. Mass spectra data were acquired on a MAT 95XL (ThermoQuest Finigan) equipped with an electron ionization (EI) or electrospray ionization (ESI) resource. Flash column chromatography (flash CC) was performed with silica gel (Woelm Pharma 60 mesh) or neutral alumina (II-III Brockmann activity, EcoChrom, 100–150 mesh). Solvents were obtained dry from a MB SPS-800 Solvent Purification System (MBraun). Products containing water were freeze-dried on a Virtis Benchtop 3.3/Vacu-Freeze connected to a Vacubrand oil-pump. The mean hydrodynamic diameter of liposomes and lipoplexes at various N/P (+/−) molar charge ratios was measured by quasi-elastic light scattering with a Zetasizer APS (Malvern Instruments, Worcestershire, UK). For each sample, the measurement was performed three times for two different batches at a temperature of 25 °C with a detection angle of 90°.

### 4.4. Synthesis

#### 4.4.1. Synthesis of *β-apo-8´-Carotenoic Acid* (**7**)

To ethyl**-**β-apo-8´-carotenoate (**6**, 2.00 g, 4.35 mmol), KOH in MeOH (25%, 10 mL) was added and stirred for 5 min. *n*BuOH (200 mL) was added and the mixture was heated to 110 °C for 2 h under N_2_, until TLC indicated full conversion to **7**. The solvent was removed under reduced pressure and H_2_SO_4_ (25%, 20 mL) was added to the residue. This mixture was stirred vigorously for 5 min; distilled water (10 mL) was added and stirring continued for 30 min. The slurry was extracted with CH_2_Cl_2_ (2 × 100 mL) and the organic phase was washed with distilled water (3 × 200 mL), dried over anhydrous Na_2_SO_4_, concentrated and dried under vacuum. Carotenoic acid **7** was obtained quantitatively and was used directly without further purification. Spectroscopic data are in accordance with literature [23].

#### 4.4.2. Synthesis of *2-(*N,N*-Dimethylamino)ethyl-β-apo-8′-carotenoate* (**8**)

β-Apo-8´-carotenoic acid (**7**, 580 mg, 1.34 mmol) and 1,1'-carbonyldi(1,2,4-triazole) (275 mg, 1.68 mmol) were dissolved in dry CH_2_Cl_2_ (75 mL) and a crystal of 4-(*N*,*N*-dimethylamino)pyridine (DMAP) was added. The mixture was stirred at room temperature under N_2_ for 1 h, until TLC indicated full conversion. Dry 2-*N*,*N*-dimethylaminoethanol (0.82 mL, 8.19 mmol) was added, and the mixture was refluxed for 5 h. The mixture was washed with distilled water (3 × 50 mL), the organic phase dried over anhydrous Na_2_SO_4_ and concentrated. The residue was purified by flash-CC on silica with CH_2_Cl_2_/MeOH gradient and recrystallized from acetone (458 mg, 68%). TLC (toluene/acetone/MeOH, 6/1/1 v/v): R_f_ = 0.50. m.p.: 130.3–131.5 °C. UV/Vis (CH_2_Cl_2_): λ_max_ = 457 nm. ^1^H-NMR: carotenoyl part in accordance with our previous work [23], 4.276 (t, 2H, H-1), 2.645 (t, 2H, H-2), 2.318 (s, 6H, H-4/H-1'); ^13^C-NMR: 62.696 (CH_2_, C-1), 57.925 (CH_2_, C-2), 45.879 (CH_3_, C4/C-1'); HRMS (ESI): C_34_H_49_NO_2_ calcd. 503.3763 (M^+^), found 503.3749.

#### 4.4.3. Synthesis of *2-(*N,N*-Dimethylamino)ethyl-β-apo-8'-carotenoate hydrobromide* (**1**)

The dimethylamine analogue **8** (458.5 mg, 0.91 mmol) was dissolved in MeOH (30 mL) and HBr (1 M in water, 2 eq., 1.82 mL, 1.82 mmol) was added. The mixture was stirred at room temperature under N_2_ for 3 h. The solvent was removed under reduced pressure and water by freeze-drying. The residue was dissolved in CH_2_Cl_2_ and crystallized in hexane giving **1** (445.8 mg, 0.76 mmol, 84%). m.p.: 162.1–164.3 °C; UV/Vis (CH_2_Cl_2_): λ_max_ = 462 nm; ^1^H-NMR: 4.541 (m, 2H, H-1), 3.568 (m, 2H, H-2), 2.995 9s, 6H, H-4/H-1'); ^13^C-NMR: 57.653 (CH_2_, C-1), 55.698 (CH_2_, C-2), 42.329 (CH_3_, C4/C-1'); HRMS (ESI): C_34_H_50_NO_2_ calcd. 504.3842 (M^+^), found 504.3817.

#### 4.4.4. Synthesis of *2-Bromoethyl-β-apo-carotenoate* (**9**)

Carotenoic acid **7** (1.00 g, 2.31 mmol), dicyclohexylcarbodiimide (DCC, 0.72 g, 3.47 mmol), DMAP (56 mg, 0.46 mmol) and 2-bromoethanol (5.77 g, 46.2 mol) were dissolved in dry CH_2_Cl_2_ and stirred at room temperature under N_2_ for 18 h. The reaction mixture was extracted with water (3 × 50 mL), dried over anhydrous Na_2_SO_4_ and concentrated. The residue was dissolved in cold acetone (10 mL) and filtered to remove the urea formed from DCC. The bromoethyl carotenoate **9** was recrystallized from acetone (1.06 g, 85%). TLC (hexane/acetone 8/2 v/v): R_f_ = 0.80; m.p.: 132.8–133.6 °C; UV/Vis (CH_2_Cl_2_): λ_max_ = 460 nm; ^1^H-NMR: 4.461 (t, 2H, H-1), 3.579 (t, 2H, H-2); ^13^C-NMR: 63.873 (CH_2_, C-1), 29.096 (CH_2_, C-2); HRMS (EI): C_32_H_43_BrO_2_ calcd. 538.24463 (M^+^), found 538.24465.

#### 4.4.5. Synthesis of *2-(*N,N,N*-Trimethyl) ethyl-β-apo-8'-carotenoate bromide* (**2**)

Bromoethyl carotenoate **9** (600 mg, 1.11 mmol) was dissolved in CHCl_3_/iPrOH/DMF (3/5/5/ v/v 50 mL) and NMe_3_ (45% in water, 5 mL) was added. The mixture was stirred at room temperature under N_2_ for 4 days. The solvents were removed under reduced pressure, the residue dissolved in CH_2_Cl_2_ (50 mL) and extracted with water (3 × 50 mL). The organic phase was dried over anhydrous Na_2_SO_4_ and concentrated. The residue was purified by flash-CC Al_2_O_3_ with a toluene/acetone/MeOH gradient and the product was isolated with 5% MeOH in acetone. The desired quaternized amine **2** was recrystallized from acetone (565 mg, 86%). TLC: (CHCl_3_/MeOH/H_2_O, 65/25/4 v/v), R_f_ = 0.30; m.p.: 230.8–231.3 °C; UV/Vis (CH_2_Cl_2_): λ_max_ = 464 nm; ^1^H-NMR: 4.670 ((b, 2H, H-1), 4.176 (b, 2H, H-2), 3.582 (s, 9H, H-4/H-5/H-6); ^13^C-NMR: 57.929 (CH_2_, C-1), 65.131 (CH_2_, C-2), 54.405 (CH_3_, C-4/C-5/C-6); HRMS (ESI): C_35_H_52_NO_2_ calcd. 518.3998 (M^+^), found 518.3988.

#### 4.4.6. Synthesis of *2-(*N*-(2-Hydroxyethyl)*, N*-Methylamino)ethyl-β-apo-8'-carotenoate* (**10**)

Carotenoic acid **7** (390 mg, 0.90 mmol), DCC (280 mg, 1.25 mmol) and DMAP (22 mg, 0.18 mmol) were dissolved in dry CH_2_Cl_2_ (5mL) and N-methyldiethanolamine (5 mL) was added. The mixture was stirred at room temperature under N_2_ for 3 days. The solution was extracted with water (3 × 50 mL), the organic layer dried over anhydrous Na_2_SO_4_ and concentrated in cold acetone (5 mL) and filtered to remove urea. Purification by flash-CC on silica with a toluene/acetone gradient eluted the product at 10% acetone. The desired ethanolamine product **10** was recrystallized from acetone (197 mg, 41%). TLC (hexane/acetone 8/2 v/v): R_f_ = 0.38; UV/Vis (CH_2_Cl_2_): λ_max_ = 456 nm; ^1^H-NMR: 4.178 (t, 2H, H-1), 2.696 (t, 2H, H-2), 2.539 (t, 2H, H-4), 3.494 (t, 2H, H-5), 2.261 (s, 3H, H-1'); ^13^C-NMR: 62.005 (CH_2_-C-1), 55.865 (CH_2_, C-2), 58.891 (CH_2_, C-4), 58.283 (CH_2_, C-5), 42.008 (CH_3_, C-1').

#### 4.4.7. Synthesis of *2-(*N*-(2-Hydroxyethyl*), N,N*-Dimethylammonium)ethyl-β-apo-8'-carotenoate iodide* (**3**)

Ethanolamine **10** (190 mg, 0.35 mmol) was dissolved in dry THF (50 mL) and MeI (3 mL) was added. The mixture was stirred at room temperature under N_2_ for 3 days and the crude product was isolated by filtration. The quaternized ethanolamine **3** was recrystallized from CH_2_Cl_2_ and hexane (124 mg, 53%). TLC (CHCl_3_/MeOH/H_2_O 70/30/3 v/v): R_f_ = 0.37; m.p.: 183.2–185.7 °C; UV/Vis (CH_2_Cl_2_): λ_max_ = 465 nm; ^1^H-NMR: 4.655 (t, 2H, H-1), 4.097 (t, 2H, H-2), 3.882 (t, 2H, H-4), 4.169 ((t, 2H, H-5), 3.469 (s, 6H, H-1'/H-1"); ^13^C-NMR: 58.036 (CH_2_-C-1), 64.210 (CH_2_, C-2), 66.640 (CH_2_, C-4), 55.761 (CH_2_, C-5), 53.142 (CH_3_, C-1'/C-1"); HRMS (ESI): C_36_H_54_NO_3_ calcd. 548.4102 (M^+^), found 548.4101.

#### 4.4.8. Synthesis of *2-(*N,N*-Di(2-hydroxyethyl)amino)ethyl-β-apo-8'-carotenoate* (**11**)

Carotenoic acid **7** (1.216 g, 2.81 mmol), DCC (870 mg, 4.22 mmol) and DMAP (70 mg, 0.56 mmol) were dissolved in dry CH_2_Cl_2_ (50 mL) and triethanolamine (10 mL) was added. The mixture was stirred at room temperature under N_2_ for 3 days. The solution was extracted with water (3 × 50 mL), the organic layer dried over Na_2_SO_4_ and concentrated under reduced pressure. The residue was dissolved in cold acetone (5 mL) and filtered to remove DCC-urea. Purification by flash-CC on silica with a toluene/acetone gradient eluted the product at 10% acetone. Diethanolamine **11** was recrystallized from acetone (937 mg, 59%). TLC: (toluene/acetone/MeOH 6/1/1/ v/v): R_f_ = 0.39; m.p.: 136.4–137.3 °C; UV/Vis (CH_2_Cl_2_): λ_max_ = 454 nm; ^1^H-NMR: 4.294 (t, 2H, H-1), 2.985 (t, 2H, H-2), 2.756 (t, 4H, H-4/H-1´), 3.625 (t, 4H, H-5/H-2´); ^13^C-NMR: 62.434 (CH_2_, C-1), 53.958 (CH_2_, C-2), 56.766 (CH_2_, C-4/C-1'), 59.819 (CH_2_, C-5/C2´); HRMS (ESI): C_36_H_53_NO_4_ calcd. 563.3975 (M^+^), found 563.3997.

#### 4.4.9. Synthesis of *2-(*N,N*-Di(2-hydroxyethyl)amino)ethyl-β-apo-8'-carotenoate hydrobromide* (**4**)

The diethanolamine analogue **11** (586 mg, 1.04 mmol) was dissolved in MeOH (30 mL) and HBr (0.1 M in water, 20.8 mL, 2.08 mmol) was added. The mixture was stirred at room temperature under N_2_ for 3 h. The solvent was removed under reduced pressure and water by freeze-drying. The residue was dissolved in CH_2_Cl_2_ and crystallized by addition of hexane giving **4** (587 mg, 90%). m.p.: 195.4–199.5 °C; UV/Vis (CH_2_Cl_2_): λ_max_ = 458 nm; ^1^H-NMR: 4.675 (b, 2H, H-1), 3.775 (b, 2H, H-2), 3.543–3.656 (s, 4H, H-4/H-1'), 4.074–4.157(b, 4H, H-5/H-2'), 9.301 (b, 1H, NH); ^13^C-NMR: 58.080 (CH_2_, C-1), 55.375 (CH_2_, C-2), 56.085 (CH_2_, C-4/C-1'), 55.701 (CH_2_, C-5/C-2´), 53.142 (CH_3_, C-1'/C-1"); HRMS (ESI): C_36_H_54_NO_4_ calcd. 564.4052 (M^+^), found 564.4053.

#### 4.4.10. Synthesis of *2-(*N,N*-Di(2-hydroxyethyl)*, N*-methylammonium)ethyl-β-apo-8'-carotenoate Iodide* (**5**)

The diethanolamine analogue **11** (309.4 mg, 0.55 mmol) was dissolved in dry THF (50 mL) and MeI (3 mL) was added. The mixture was stirred at room temperature under N_2_ for 24 h and the crude product was separated by filtration. The quaternized ammonium cation **5** was recrystallized from CH_2_Cl_2_ and hexane (308 mg, 80%). TLC (CHCl_3_/MeOH/H_2_O, 70/30/3 v/v): R_f_ = 0.33; m.p.: 199.8–203.1 °C; UV/Vis (CH_2_Cl_2_): λ_max_ = 464 nm; ^1^H-NMR: 4.678 (b, 2H, H-1), 4.128 (b, 2H, H-2), 3.924 (s, 4H, H-4/H-1´), 4.213 (b, 4H, H-5/H-2'), 3.474 (s, 3H, H-1"); ^13^C-NMR: 58.192 (CH_2_, C-1), 62.882 (CH_2_, C-2), 65.076 (CH_2_, C-4/C-1'), 55.861 (CH_2_, C-5/C-2'), 51.476 (CH_3_, C-1"); HRMS (ESI): C_37_H_56_NO_4_ calcd. 578.4209 (M^+^), found 578.4208.

### 4.5. Particle Formulation Methods

*Ethanolic stock solutions.* Ethanolic stock solutions were made for each individual cationic lipid and co-lipid by dissolving a known amount of lipid in dichloromethane in a round-bottom flask. The dichloromethane solutions were placed on a rotary evaporator for two hours to obtain a film. The film was dissolved in a known amount of anhydrous alcohol in order to get 1 mM stock, and once dissolved the alcohol stock was stored at −80 °C.

*Liposome formulations.* Hydrated liposomes composed of cationic lipid/DOPE (3:2 molar/molar) were prepared with the cationic carotenoid lipids **1**–**5**. Commercially available cationic lipids were used to prepare two types of control liposomes: EPC as a non-rigid lipid and DC-Chol as a rigid lipid. Required amounts of each alcohol solution of lipid and co-lipid were combined and evaporated under reduced pressure to form thin films. These last were hydrated with a known amount of sterile water, followed by sonication to give a 2 mM final solution of hydrated stocks. These liposomal stock solutions were stored overnight at 4 °C. Before use, the hydrated stocks were warmed to 37 °C for 5 min in a water bath, then sonicated for 30 min.

*Lipid/siRNA lipoplexes.* Lipid/siRNA lipoplexes were formulated by adding 54 µL of OPTI-MEM^®^ (Gibco Cell Culture, Carlsbad, CA, USA) with 6 µL of siRNA (either GL2 or control) to give siRNA aliquots. Liposomes were diluted in OPTI-MEM^®^ to get 60 µL Aliquots of desired molar concentration. SiRNA aliquots were added to the microcentifuge tubes containing the diluted liposomes, mixtures were pipette thoroughly and incubated for 20 min at room temperature before adding 180 µL of OPTI-MEM^®^ to each formulation and applying them on the cells as described in the assay section.

### 4.6. Bioassay Methods

*Cell culture.* Cationic lipid mediated transfection of siRNA duplex (GL2) for specific knockdown of the luciferase transcript as well as of validated control siRNA, was performed using HR5-CL11 cells following standard methods. Briefly, HR5-CL11 cells were grown in DMEM media supplemented with 10% fetal calf serum and 100 U/mL of penicillin/streptomycin and the equivalent of 1 µg/mL doxycycline. Cells were seeded 24 h before transfection onto opaque and transparent 96-well plate at a density of 10^4^ cells per well and incubated with a 5% CO_2_ atmosphere at 37 °C. Cells were grown to 80% confluence before being washed with PBS and incubated with 50 µL of each lipid-siRNA complex in triplicate for 4 h at 37 °C. The complexes were then removed by aspiration and the cells washed with PBS before adding 100 µL of DMEM media containing 2 µg/mL of doxycycline to each well. Cells were left to incubate additional 44 h. Following the incubation cells were used for the assays of luciferase, total proteins and cytotoxicity following the bellow mentioned protocols.

*Luciferase knockdown assay.* Forty-eight hours after the application of lipoplexes, treated cells in the opaque 96-well plates were washed with PBS, and lysed by adding 50 µL of Glo-Lysis^TM^ buffer to each well. After a 15 min incubation period at room temperature, 50 µL of Bright Glo^TM^ working solution, prepared according to the manufacturer’s directions (Promega, Madison, WI, USA) were added to each well and mixed by pipetting. Luminescence was then read on a Victor Envision high throughput plate reader.

*Total protein (BCA) assay.* Total protein content was measured using Pierce^®^ BCA Protein Assay (Pierce Biotechnology, Rockford, IL, USA). Forty-eight hours after the application of lipoplexes, treated cells in the transparent 96-well plate were washed with PBS, then 10 µL of passive lysis buffer (Promega) was added and the plate was incubated at room temperature for 5 min with gentle shaking. BCA working reagent (200 µL), prepared according to the manufacturer’s directions, was then added to each well and gently mixed by pipetting. Plates were incubated at room temperature for 1 h then read at 562 nm on a Victor Envision high-throughput plate reader. A calibration curve generated from a bovine serum albumin standard solution was used to determine cellular protein content per well.

*Cytotoxicity assay.* The cellular toxicity of the various lipoplex formulations was evaluated using the HR5-CL11 cell line through an MTS assay. Forty-eight hours after the application of lipoplexes, treated cells in the transparent 96 well plate were washed with PBS, and 20 µL of CellTiter96^®^ solution (CellTiter96^®^ Aqueous One Solution Cell Proliferation Assay) was added and the cells were incubated further for 1 h at 37 °C. The absorbance of converted dye, which correlates with the number of viable cells, was measured at 490 nm using a Victor Envision high throughput plate reader. The percentage of viable cells was calculated as the absorbance ratio of treated to untreated cells.

## 5. Conclusions

The literature clearly indicates that cationic lipid gene transfer vectors with two hydrophobic chains are generally more active than those with a single hydrophobic chain [32]. Our preliminary findings in this study suggest that our novel, single-chain rigid cationic carotenoid lipids are able to complex and deliver siRNA across the cell membrane of eukaryotic cells. However, the results from this preliminary study comparing our single-chain rigid lipids with the rigid control lipid DC-Chol do not support our hypothesis of a correlation between liposome packing and siRNA delivery efficiencies.

Of the five single-chain carotenoid lipids investigated, lipids **1**, **2**, **3** and **5** displayed good knockdown efficiency with HR5-CL11 cells at defined (+/−) molar charge ratios. In addition, lipid **1** exhibited the lowest levels of cytotoxicity with cell viability greater than 80% at all (+/−) molar charge ratios studied, exceeding the cell viability of both control lipids, DC-Chol and EPC. These novel, single-chain rigid carotenoid-based cationic lipids represent a new class of transfection vectors with good cell tolerance accompanied with encouraging *in vitro* luciferase knockdown activity in HR5-CL11 cells. Our ongoing efforts are focused on the enhanced efficiency of these single-chain transfection vectors through modification at the lipid headgroup, counter ion and lipoplex formulation.
